# Replication-Competent Influenza A Viruses Expressing Reporter Genes

**DOI:** 10.3390/v8070179

**Published:** 2016-06-23

**Authors:** Michael Breen, Aitor Nogales, Steven F. Baker, Luis Martínez-Sobrido

**Affiliations:** Department of Microbiology and Immunology, University of Rochester School of Medicine and Dentistry, 601 Elmwood Avenue, Rochester, NY 14642, USA; michael.breen24@gmail.com (M.B.); Aitor_nogales@urmc.rochester.edu (A.N.): steven.baker@wisc.edu (S.F.B.)

**Keywords:** recombinant influenza A virus, plasmid-based reverse genetics, virus rescue approaches, reporter genes, fluorescence, luminescence, replicating-competent reporter-expressing influenza A virus

## Abstract

Influenza A viruses (IAV) cause annual seasonal human respiratory disease epidemics. In addition, IAV have been implicated in occasional pandemics with inordinate health and economic consequences. Studying IAV, in vitro or in vivo, requires the use of laborious secondary methodologies to identify virus-infected cells. To circumvent this requirement, replication-competent IAV expressing an easily traceable reporter protein can be used. Here we discuss the development and applications of recombinant replication-competent IAV harboring diverse fluorescent or bioluminescent reporter genes in different locations of the viral genome. These viruses have been employed for in vitro and in vivo studies, such as the screening of neutralizing antibodies or antiviral compounds, the identification of host factors involved in viral replication, cell tropism, the development of vaccines, or the assessment of viral infection dynamics. In summary, reporter-expressing, replicating-competent IAV represent a powerful tool for the study of IAV both in vitro and in vivo.

## 1. Introduction

### 1.1. Influenza A Virus

**Figure 1:**
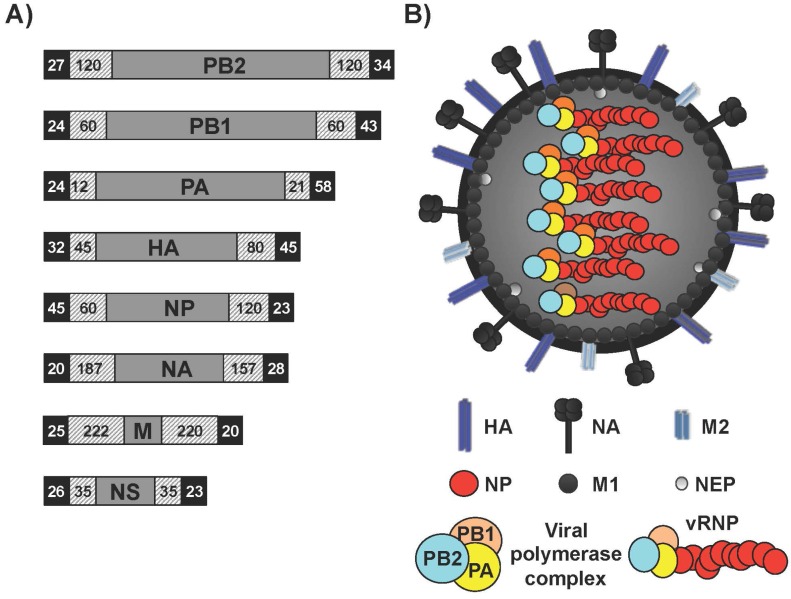
**Influenza A virus (IAV) genome organization and virion structure.** (**A**) Genome organization: The eight single-stranded, negative-sense, viral (v)RNA segments PB2, PB1, PA, HA, NP, NA, M and NS of IAV are indicated. Black boxes at the end of each of the vRNAs indicate the 3′ and 5′ non-coding regions (NCR). Hatched boxes indicate the packaging signals present at the 3′ and 5′ ends of each of the vRNAs that are responsible for efficient encapsidation into nascent virions. Numbers represent nucleotide lengths for each of the NCR and packaging signals; (**B**) Virion structure: IAV is surrounded by a lipid bilayer containing the two viral glycoproteins hemagglutinin (HA), responsible for binding to sialic acid-containing receptors; and neuraminidase (NA), responsible for viral release from infected cells. Also in the virion membrane is the ion channel matrix 2 (M2) protein. Under the viral lipid bilayer is a protein layer composed of the inner surface envelop matrix 1 (M1) protein, which plays a role in virion assembly and budding; and the nuclear export protein (NEP) involved in the nuclear export of the viral ribonucleoprotein (vRNP) complexes. Underneath is the core of the virus made of the eight vRNA segments that are encapsidated by the viral nucleoprotein (NP). Associated with each vRNP a complex is the viral RNA-dependent RNA polymerase (RdRp) complex made of the three polymerase subunits PB2, PB1 and PA that, together with the viral NP are the minimal components required for viral replication and transcription.

Influenza A viruses (IAV) are enveloped viruses within the family *Orthomyxoviridae* [1]. The genome of IAV contains eight single-stranded, negative-sense viral RNA (vRNA) segments [1] (Figure 1A). The vRNAs contain a long central coding region that is flanked at both termini by non-coding regions (NCRs), which serve as promoters to initiate replication and transcription by the viral heterotrimeric polymerase complex [1,2,3]. vRNAs reside within the virion as viral ribonucleoprotein (vRNP) complexes bound to a viral polymerase and many copies of nucleoprotein (NP) (Figure 1B). IAV are important pathogens that exert a dramatic impact on public health and the global economy [4] and cause annually recurrent epidemics, which result in approximately three to five million cases of severe illness and 250,000 to 500,000 deaths worldwide [5]. IAV are classified on the basis of the antigenic properties of the enveloped glycoproteins hemagglutinin (HA) and neuraminidase (NA), into 18 HA (H1–H18) and 11 NA (N1–N11) subtypes [6,7]. The HA protein is critical for binding to cellular receptors and fusion of the viral and endosomal membranes [8,9]. Additionally, infection with IAV results in protective immunity mediated, at least in part, by antibodies against the viral HA, which is the key immunogen in natural immunity and vaccine approaches. The NA protein cleaves sialic acid moieties from sialyloligosaccharides and facilitates the release of nascent virions [10,11]. Importantly, NA is a major target for antiviral drugs, such as oseltamivir, that block the aforementioned cleavage and prevent viral dissemination to prevent further infection [12,13].

The replication and transcription process of influenza vRNAs are carried out by NP and the three polymerase subunits, an acidic (PA) and two basic (PB1 and PB2) proteins, which are encoded by the three largest vRNA segments [1]. Unlike many RNA viruses, influenza viral genome replication and transcription occurs in the nucleus of infected cells [14]. Newly synthesized vRNP complexes are then exported from the nucleus to the cytoplasm by the nuclear export protein (NEP) and the matrix protein 1 (M1), and are assembled into virions at the plasma membrane [1]*.* The small IAV genome is able to transcribe multiple viral genes from single segments through multiple mechanisms. These mechanisms include alternative splicing of viral mRNAs (M and NS segments), non-canonical translation, non-AUG initiation, or ribosomal frameshifting [1,15,16,17,18,19]. Moreover, to extend the coding capability of the viral genome, IAV encode proteins containing more than one function during virus infection. A well-studied multifunctional IAV protein is the non-structural protein 1 (NS1), which is expressed at very high levels in infected cells and is a determinant of virulence that functions in several ways to defeat cellular innate antiviral mechanisms [20]. NS1 is encoded on a collinear mRNA derived from vRNA segment eight (NS), which upon splicing results in the synthesis of NEP [21].

Although the natural reservoirs of IAV are wild waterfowl and shorebirds, IAV expand their host range to many avian and mammalian species through undefined adaptive processes involving mutation and genome reassortment [22,23], and this cross-species jumping characteristic allows the generation of potentially pandemic strains. In addition, antigenic drift occurs when the virus accumulates mutations that preclude binding by pre-existing antibodies, producing variant viruses that can escape immunity. IAV of three HA subtypes (H1, H2 and H3) thus gained the ability to be transmitted efficiently among humans [24]. In addition, IAV of the H5, H7 and H9 subtypes are also thought to represent pandemic threats because they have crossed the species barrier and infected humans [25,26,27,28]. Given the persistent threat posed by IAV infections, accelerating the development of novel countermeasures against IAV infections and increasing the biological understanding associated with viral infections are imperative.

Current available options to counter IAV include both vaccines and antivirals [12,29]. Only two classes of antivirals are approved for IAV that target either the ion channel function of the matrix 2 (M2) protein or the neuraminidase function of the NA protein [12]. However, these antiviral compounds have problems in terms of safety and the emergence of viral resistance [12,30,31]. Vaccines, due to the induction of sterilizing immunity, are the primary means to prevent IAV infections. However, currently available vaccines have moderate efficacy that changes seasonally [32]. Moreover, to generate vaccines against highly pathogenic IAV, as in the case of a pandemic outbreak, requires time. Therefore, developing new antiviral strategies to combat IAV infections are urgently needed. Current and traditional technologies to identify antivirals against IAV have been extensively reviewed [33]. This review will focus on the application and limitations of replication-competent IAV harboring fluorescent and/or luminescent reporter genes. Through better knowledge of the influenza virus genome, most importantly the identification of vRNA packaging signals [34,35,36,37], it has become possible to engineer replication-competent IAV encoding exogenous genes [38,39,40,41]. Stable incorporation of foreign genes in replication-competent, reporter-expressing IAV allows for effective tracking of viral infection in vitro and in vivo enabling a robust quantitative readout. This readout can be used with high throughput screenings (HTS) and to assess viral infection in tissue culture cells and animals models without the use of secondary approaches to identify the presence of the virus.

### 1.2. Comparison of Fluorescent and Luciferase Reporter Genes

The major advantage of using recombinant, replication-competent IAV is their flexibility to support the presence of reporter genes, such as fluorescence or luciferase proteins, in different viral segments. These reporter genes provide a good readout of viral replication and are compatible with HTS settings [33,39,42,43]. Moreover, reporter genes have a noteworthy role in multiple applications, both in vitro and in vivo. An ideal reporter gene encodes a protein whose activity can be detected with high sensitivity above any endogenous background and is amenable to assays that are sensitive, quantitative, and reproducible. In addition, reporter proteins can be detected directly by its inherent characteristics, such as fluorescence or enzymatic activity, as well as indirectly with antibody-based assays like Western blot. Although there are multiple reporter genes that can be used, this review will focus on replication-competent recombinant IAV harboring fluorescence or luciferases reporters, two categories of proteins that glow. Both types of systems (fluorescence and bioluminescence) create photons through energy transitions from excited states to their corresponding ground states. However, they differ in how the excited states are generated. The glow mechanism for fluorescent proteins is generated by first absorbing energy of one color light (excitation), and then emitting energy as a different wavelength [42]. On the other hand, the bioluminescence glow results from exothermic chemical reactions [44].

The first bioluminescent reporter identified was named aequorin, a calcium-activated photoprotein from the *Aequorea victoria* jellyfish [45]. However, the discovery of green fluorescent protein (GFP) in the early 1960s, with the gene first being cloned in 1992, ultimately heralded a new era in molecular biology [42]. GFP was identified as a protein that lacked the bioluminescent properties of aequorin, but was able to generate fluorescence when illuminated with UV light [42]. More recently, fluorescent and bioluminescence proteins from other species have been identified, and mutant variants have been developed with different glow properties, resulting in rapid expansion of the color spectrum [43]. Importantly, newly developed technologies to excite or detect emission has helped expand a range of applications [46].

Cloning of the *luc* gene from the firefly *Photinus pyralis* provided the first luciferase reporter system with widespread utility in mammalian cells [47]. The luciferase family of enzymes generates bioluminescent signals through mono-oxygenation of luciferin (substrate); utilizing ATP and O_2_ as co-substrates with luciferin, luciferase catalysis produces light [44,46]. Luciferase enzymes isolated from different animal species have different variability in light emission, sensitivities, and emission duration times that accommodate different experimental designs [44,46]. Multiple luciferase enzymes can further be combined for multiplex analyses, including in vivo imaging [46]. Moreover, new secreted versions of luciferases [48] and shorter versions of luciferase genes [49] have been described to facilitate detection of reporter gene expression upon viral infection.

Properties of reporter genes must be considered on a per experiment basis because different genes will serve different purposes and choosing the best reporter gene assay depends on the type of study (Table 1) [39]. For example to observe localization, fluorescent genes like GFP are most convenient [38,42]. However, for quantitative purposes, luciferases are more useful [50,51]. Whole-body imaging is increasingly used in mice or other small laboratory animals [46]. For this purpose, luciferase reporters are preferred over fluorescent proteins because fluorescence requires excitation light to travel to the location of the fluorescent probe, while luciferase substrates can be administered systemically [46]. For fluorescent targets in vivo, excitation light is scattered from tissue above the plane of the target, which reduces the intensity. Moreover, the sensitivity and specificity of fluorescence imaging are frequently disturbed by tissue autofluorescence, resulting in substantial background [42,43]. Problems arising from tissue penetrance and autofluorescence are reduced when fluorescent proteins are visualized ex vivo. Bioluminescence reporters also have limitations, since the production of light requires the presence of all components involved in the oxidation reaction. Although some reagents like ATP or O_2_ exist in tissues, the concentrations can vary by anatomical location and the physiological condition of the animal [51]. Moreover the substrate (luciferin) must be injected into the animal to generate the bioluminescent signal [51,52]. It should be noted that advances in instruments used for molecular imaging are improving in sensitivity and resolution, helping to minimize some of the aforementioned limitations with both types of reporter genes.

## 2. Generation of Replication-Competent IAV Harboring Reporter Genes

IAV segmented genome allows the opportunity to tag various gene segments with fluorescent or luminescent reporters, thus allowing for visual and/or quantitative observation of reporter gene expression. Several caveats must be considered when designing IAV encoding reporter genes. First, the virus segments are small (~0.9–2.4 kb in length) and do not tolerate large insertions. Second, adding a reporter gene in the 3′ or 5′ end of the viral segment disrupts packaging signals located at the end of each viral RNA that are required for efficient virion assembly. Third, it is important to evaluate the stability of the inserted reporter gene since some replicating-competent IAV has been described to easily lose the inserted reporter gene. The causes associated to the reporter instability are not totally understood, however can be related with the size or the nature of the inserted foreign sequence. To overcome these hurdles, multiple strategies have been employed to rescue recombinant IAV harboring reporter genes, leading to an abundance of reporter-containing IAV (Table 2, Table 3, Table 4, Table 5, Table 6 and Table 7).

### 2.1. Reporter-Expressing IAV Containing the Foreign Gene in the PB2 Segment

**Figure 2:**
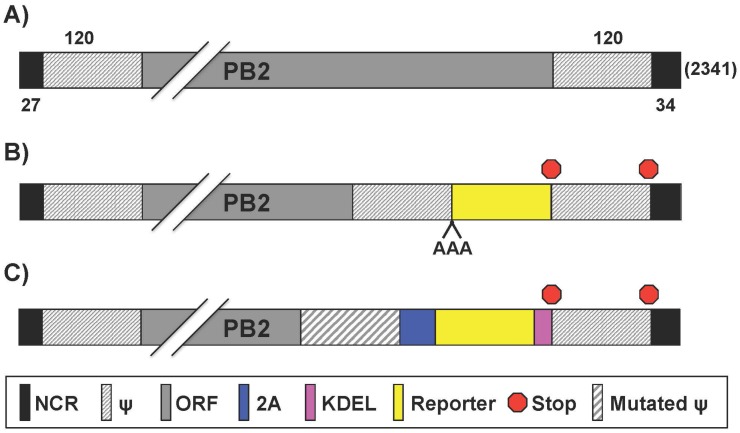
**PB2 reporter influenza A viruses:** Schematic representation of the PB2 segment from wild type (WT) (**A**) and reporter (**B**,**C**) IAV. Gene segments all contain non-coding regions (NCR), packaging signals (ψ) and open reading frames (ORF) for gene replication/transcription, virion incorporation, and protein expression, respectively. Nucleotide lengths for the NCR, ψ, and PB2 segment are indicated; (**B**) PB2 fusion proteins: Reporter genes were fused to native PB2 ORF with a triple alanine (AAA) spacer; (**C**) Bicistronic transcription of PB2 and reporter gene: Insertion of the 2A autocleavage site separates PB2 from reporter gene. Packaging signals encoding the 3′ terminus of PB2 were mutated to minimize interference with native ψ, which are duplicated at the 3′ NCR-proximal region. KDEL (lysine-aspartic acid-glutamic acid-leucine) signal sequence was inserted for endoplasmic reticulum-retention of the reporter gene. Packaging signals were duplicated after protein stop transcription signal and before the 3′ NCR terminus.

IAV segment 1 (Figure 2A) encodes for the PB2 protein that plays an important role in viral genome transcription initiation by generating 5′-capped RNA fragments from cellular pre-mRNA molecules, which serve as primers for viral transcription [53,54]. PB2 also modulates vRNP assembly and is thought to contribute to viral replicase or transcriptase activity [14]. Finally, IAV PB2 is a major host range and virulence determinant [55,56,57].

Studies to examine intracellular vRNP trafficking were previously limited due to the difficulty of visualizing their movement in living cells. To solve this, Avilov et al. [58] utilized “split-GFP” [59], where the 16 C-terminal amino acids (aa) of GFP were fused to PB2 (Figure 2B), and GFP reconstitution occurs in *trans*-complementing transiently transfected cells [58] (Table 2). The GFP-tagged virus in the backbone of influenza A/WSN/1933 H1N1 (WSN) was deemed “WSN-PB2-GFP11” [58]. This virus had a similar, albeit slightly diminished, growth kinetic and plaque phenotype compared to wild-type (WT) WSN virus in both parental and *trans*-complementing Madin-Darby canine kidney (MDCK) cells [58]. While it does propagate efficiently in vitro, a distinct disadvantage is that transfected cells are required to observe fluorescence, which is simplified by generating stable cell lines expressing the GFP *trans*-complementing domain [58]. In addition, the generation of mice expressing the same GFP domain, either in target tissues or constitutively, could be used for in or ex vivo analysis of viral infection. Avilov et al. used WSN-PB2-GFP11 to monitor trafficking of vRNPs during infection using dynamic light microscopy [58]. Live imaging of cells infected with a split-GFP-based virus demonstrated that over the course of infection, vRNPs accumulated pericentriolarly, followed by a wide distribution throughout the cytoplasm and an accumulation at the plasma membrane. Occasional quick movements of vRNPs were also detected in the cytoplasm and reported to be actin- and microtubule-dependent. These results were in agreement with previous observations of fixed cells [59,60]. Furthermore, Avilov et al. observed vRNP association with Rab11 [61] (Table 2), a host protein involved in cellular vesicle trafficking [62]. Their findings reiterate previous reports that vRNPs accumulate in Rab11 containing particles [63,64,65]. The authors used fluorescence resonance energy transfer microscopy to suggest a direct vRNP:Rab11 interaction and proposed that vRNPs traffic through the cytoplasm with recycling endosomes via Rab11 interactions [61].

Influenza polymerase function is tightly regulated by protein-protein interactions, and the subunits do not tolerate large foreign protein additions. The insertion of viral 2A peptides has been extensively used to generate replication-competent IAV containing foreign sequences [40,41,48,52,66,68,69,70,71,72,73]. Viral 2A sequences mediate co-translational “ribosome skipping” or protein cleavage to separate two distinct polypeptides [74]. Equimolar amounts of collinear transcripts are therefore expressed from a single mRNA separated by 2A under the control of a single viral promoter. Heaton et al. [66] cloned the Gaussia luciferase (Gluc) gene into the C-terminal end of PB2 and separated the viral open reading frame (ORF) from the reporter via a foot-and-mouth disease virus (FMDV) 2A peptide sequence (Figure 2C, Table 2). Importantly, the addition of a foreign sequence at the end of the vRNA disrupts the packaging signals needed to assemble progeny virions [1]. To overcome packaging restrictions, the complete 5′ packaging signal of PB2 was duplicated after Gluc, upstream of the 5′ NCR. Moreover, silent mutations were introduced into the original 5′ packaging signals in the PB2 ORF, to eliminate the original packaging signals. Additionally, an endoplasmic reticulum (ER) retention sequence (KDEL) was added to the C-terminus of Gluc to prevent secretion, and the virus was rescued in the backbone of influenza A/Puerto Rico/8/1934 (PR8) [66]. In embryonated chicken eggs, this PR8-Gluc had 1 log lower replication levels compared to WT PR8, and the recombinant virus was stable for at least four serial passages in eggs [66]. A major interest in the IAV field is the characterization of antibodies that bind to the conserved “stalk” region of the HA glycoprotein. This domain is much less variable than the “head” region, and therapeutics targeting this domain potentially have the ability to cross-protect against multiple variants and subtypes of IAV [75,76]. PR8-Gluc was used in vivo to characterize the therapeutic potential of two stalk-reactive monoclonal antibodies (MAbs) GG3 and KB2 [77]. These stalk-reactive MAbs bind to many H1 and H5 IAV and exhibit neutralization activities in plaque reduction assays [78]. The ability of the broadly neutralizing GG3 and KB2 to impede PR8-Gluc infection in the lungs was examined by passive transfer experiments [79]. To this end, mice were given the GG3 and KB2 antibodies 2 h before being infected with a 5× mouse lethal dose-50 (MLD_50_). The studies showed no morbidity or mortality of mice receiving the antibody therapies [66]. These MAbs also protected against lethal challenge with influenza A/Netherlands/602/2009 (H1N1) and influenza A/Vietnam/1203/2004 (H5N1) in mice [66]. PR8-Gluc had a MLD_50_ of ~5000 plaque forming units (PFU), approximately 50–100 times less lethal than WT virus (MLD_50_ of ~50) [66], which suggests PR8-Gluc recapitulates a PR8 WT-like virus life cycle in vivo.

Yan et al. established a HTS protocol for the simultaneous identification of pathogen- and host-targeted hit candidates against either respiratory syncytial virus (RSV) or IAV [67]. To this end, the authors generated a recombinant WSN-Gluc (Figure 2C, Table 2), which was used with a recombinant RSV-firefly. The dual-pathogen protocol using replication-competent recombinant viruses shows superior cost and resource effectiveness. Moreover, the screening agents used in this new approach, IAV and RSV, are clinically important human respiratory pathogens.

### 2.2. IAV Containing a Viral Polymerase Subunit Fused to the Reporter Gene

**Figure 3:**
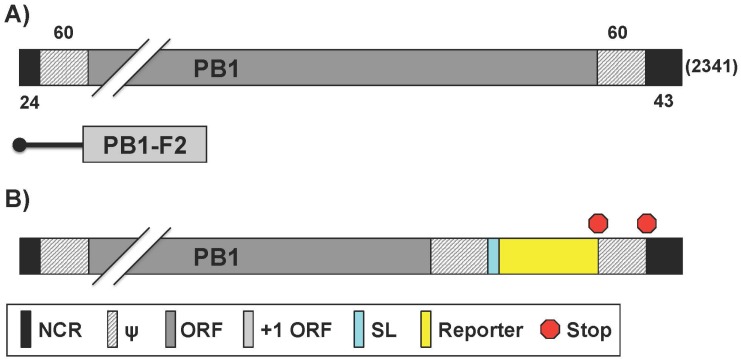
**PB1 reporter influenza A viruses:** Schematic representation of the PB1 segment from WT (**A**) and reporter (**B**) viruses as described in Figure 1. Influenza A WT PB1 viral segment encodes for both PB1 and PB1-F2 in the +1 ORF via an alternative start codon. Nucleotide lengths for the NCR, ψ, and PB1 segment are indicated; (**B**) PB1 fusion protein: Reporter genes were fused to native PB1 ORF with a short linker (SL). Packaging signals were duplicated after protein stop transcription signal and before the 3′ NCR terminus.

A precise mapping of pathogen–host interactions is essential for a comprehensive understanding of the processes of infection and pathogenesis. Interactome studies have used multiple approaches to identify and characterize protein interactions. For instance the use of yeast two-hybrid screens, which for animal viruses, does not reflect the pathogen’s microenvironment. Tandem affinity purification and mass spectrometry are also used, but this approach cannot distinguish direct from indirect interactions. New technologies are thus needed to improve the mapping of pathogen–host interactions, including IAV. Munier et al. generated a set of recombinant IAV that contain a fragment (Gluc1 or Gluc2) of a split Gluc fused to the C-terminus of PB1, PB2, or PA (vP-Gluc1 or vP-Gluc2) in the WSN backbone [80] (Figure 3, Table 3). To reconstitute Gluc activity, a cell must be co-infected by two viruses that, in combination, produce Gluc1 and Gluc2 [80]. Despite moderate attenuation in vitro relative to the WT virus, the viruses expressing a viral fusion protein (vP-Gluc1 or vP-Gluc2) were replication-competent, with the vP-Gluc1 viruses showing higher titers than their vP-Gluc2 counterparts upon multi-cycle amplification on MDCK cells [80]. The authors then used the split Gluc viruses to demonstrate a dose-dependent reduction of luciferase reconstitution in the presence of ribavirin or nucleozin, but not in the presence of amantadine, consistent with previously published data for WSN [81,82]. It is important to note that luciferase activity can be reduced by inhibiting viral protein-protein interactions or by reducing viral proteins abundance. The split Gluc viruses could thus be used with compound libraries, or knockdown or overexpression assays to identify host factors that affect viral replication, RNP assembly, or inhibitors of virus replication [80]. Munier et al. modified the experimental parameters to detect binary interactions between IAV polymerase and host proteins, whereby a single Gluc1-tagged virus was used to infect cells transfected with Gluc2-fused host proteins [80]. The assay detected viral–host protein–protein interactions within their exploratory set [80]. Among the host factors identified were those involved in the nuclear import pathway, components of the nuclear pore complex such as nucleoporin 62 (NUP62) and mRNA export factors such as nuclear RNA export factor 1 (NXF1), RNA binding motif protein 15B (RMB15B), and DDX19B [80].

### 2.3. Reporter IAV Containing a Recombinant PA Segment Harboring the Foreign Gene

**Figure 4:**
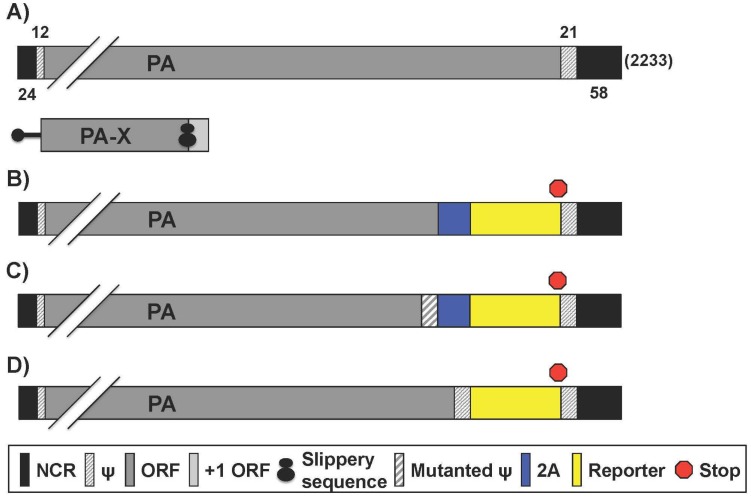
**PA reporter influenza A viruses:** Schematic representation of the PA segment from WT (**A**) and reporter (**B**–**D**) influenza A viruses as described in Figure 1. Influenza A WT PA gene segment encodes for both PA and PA-X, which shares the N-terminal amino acids with PA but the C-terminus is in the +1 ORF via ribosomal frame shift. Nucleotide lengths for the NCR, ψ, and PA segment are indicated; (**B**,**C**) Bicistronic transcription of PA and reporter gene: Insertion of the 2A autocleavage site separates PA from reporter gene. Packaging signals encoding the 3′ terminus of PA were WT (**B**) or mutated (**C**) to minimize interference with native ψ, which are duplicated at the 3′ NCR-proximal region; (**D**) PA fusion protein: Reporter genes were fused to native PA ORF. Packaging signals were duplicated after the protein stop transcription signal, before the 3′ NCR terminus.

NanoLuc (NLuc) is a small molecular weight (19 kDa) luciferase, which has a light output 150 times greater than other popular luciferases like Renilla and Firefly [49]. Because of these advantages, Tran et al. described the generation of a recombinant WSN IAV that expresses NLuc from the PA segment, using a 2A autocleavage sequence [52] (Table 4). IAV segment 3 (Figure 4A) encodes the polymerase subunit PA that has previously been shown to tolerate fusions to its C terminus without disrupting polymerase function [83]. Moreover, as compared to the other polymerase subunits, IAV PA has minimal packaging sequences at the 3′ and the 5′ end [1,84]. PA is structurally required for polymerase activity, possesses endonuclease activity to cleave host capped pre-mRNAs, and is required for nuclear accumulation of PB1 [14]. The PA segment was altered such that PA-2A-NLuc was followed by a 50 nucleotide (nt) repeat of the 3′ packaging signal; and the segment was referred to as PA-2A-NLuc50, or PATN [85] (Figure 4B). Another virus was generated (PA-SWAP-2A-NLuc50 or PASTN) that differed from PATN by introducing 18 silent mutations within the 47 terminal nt of the PA coding sequence, possibly relieving competition of multiple packaging signals to achieve stable reporter gene maintenance over repeated passaging [52] (Figure 4C). The authors showed that these NLuc WSN viruses (PATN and PASTN) replicate with WT properties in culture and in vivo*,* and possesses remarkably similar pathogenicity and lethality in mice [52]. The WSN PATN virus was then used to investigate the dissemination of IAV in mouse lungs using an in vivo imaging system. WSN PATN viruses encoding either human-signature PB2 K627 or the avian-signature PB2 E627 were used in vivo to assess host range determinants of viral dissemination in living mice [86]. As expected, mice infected with WT (PB2 K627) WSN PATN lost weight and displayed robust bioluminescence that increased over time [86]. In contrast, the WSN PATN virus encoding avian-signature PB2 E627 was severely restricted. Infected mice showed little weight loss and bioluminescence was near background levels throughout the experiment [86]. Thus, the reporter virus faithfully recapitulated the known polymerase-mediated host range restriction and could therefore also be used with newly isolated IAV strains to determine their host range and the role of species-specific adaptive mutations. PATN and PASTN virus infection is therefore a viable model for WSN infection in vitro and in vivo. Similarly, Tran et al. used this system with multi-modal bioluminescence and positron emission tomography-computed tomography (PET/CT) imaging to evaluate the effect of oseltamivir treatment in viral load, dissemination and inflammation in mice [50] (Table 4).

A similar approach was also used by Karlsson et al. to generate a PA-2A-NLuc IAV in the influenza A/California/04/2009 pandemic H1N1 (pH1N1) backbone [69] (Table 4). This recombinant virus (pH1N1-PA-NLuc) was used in a transmission study in ferrets. pH1N1-PA-NLuc had WT-like kinetics both in vitro and in vivo, and was transmissible by direct and respiratory contact [69]. Titers of pH1N1-PA-NLuc were determined by both bioluminescence and tissue culture infective dose 50 (TCID_50_) and were found to be nearly identical [69]. A benefit of using pH1N1-PA-NLuc is that measuring bioluminescence reduced the turnaround time of titer determination by 54 h [69].

Spronken and Short et al. also generated several recombinant replication-competent IAV harboring reporter genes in the PA segment [73] (Table 4). To optimize the strategy to make an IAV expressing a reporter gene, different constructs were cloned using the near-infrared fluorescent protein (iRFP) [73]. Firstly, a construct consisting of the PA 5′ untranslated region (UTR), the PA ORF without stop codon, a short linker, the 2A sequence, iRFP and the 3′ UTR was produced [73]. This construct was then further modified by inserting a duplication of the packaging region (dPR) [73]. Finally, two or three mutations in the promoter region (2UP and 3UP) were also introduced [73]. The duplication of the packaging region was essential to rescue iRFP reporter virus efficiently, whereas introduction of the 3UP mutation did not result in virus production [73]. On the other hand, the 2UP_PA_iRFP_dPR construct was the only one that resulted in recombinant virus expressing iRFP in vitro, although the virus titers were lower than that of WT [73]. A reduction in virus titer was also observed when the 2UP mutation was introduced into the WT PA gene segment [73]. The 2UP_PA_iRFP_dPR cloning strategy was later used to insert different reporters into PR8, including enhanced GFP (eGFP), far-red fluorescent protein (fRFP), Gluc and FFluc (Table 4). The levels of virus replication, reporter expression and stability of the reporter were evaluated. This strategy was then used to generate eGFP-expressing viruses in the backbone of influenza A/Netherlands/602/2009 H1N1, or the highly pathogenic avian influenza (HPAI) A/Indonesia/5/2005 H5N1, and A/Anhui/1/2013 H7N9 [73,88]. 2UP_PA-Gluc_dPR had the greatest reporter stability tested followed by 2UP_PA-eGFP_dPR. 2UP_PA-fRFP_dPR, _iRFP, and _FFLuc lost considerable reporter activity after four or five passages in vitro [73]. Further optimization, which consisted of shortening the duplicated packaging signal from 149 nt to 50 nt (2UP_PA-eGFP_sPR), had limited impact on viral replication but did enhance eGFP expression versus 2UP_PA-eGFP_dPR. When evaluating infection using an in vivo imaging system (IVIS), 2UP_PA-Gluc_sPR, _eGFP, and _fRFP showed strong signals in mouse lungs whereas 2UP_PA-iRFP_dPR did not [73]. The authors hypothesized that this is due to 2UP_PA-iRFP_dPR’s low reporter expression and not the loss of the reporter gene in vivo since MDCK cells infected with lung homogenates became fluorescent [73]. Due to strong signal output, reporter stability, and WT-like replication characteristics, 2UP_PA_eGFP_sPR was chosen as a model for further studying aspects of IAV infection, such as detecting morphological changes in infected cells by fluorescence and electron microscopy (EM) [73]. Green fluorescent (infected) MDCK cells were examined using EM and found to have microvillar projections with virus-like particles budding from these structures [73]. Finally, 2UP_PA_eGFP_sPR and _dPR pH1N1 and HPAI H5N1 and H7N9 viruses were rescued and characterized in vitro [73]. Each of the reporter viruses rescued grew ~2 logs less than WT virus in vitro [73]. Importantly, the H5N1 and H7N9 eGFP reporter viruses exhibited fluorescent stability for up to four serial passages [73]. Conversely, 2UP_PA_eGFP_sPR pH1N1 lacked stability while the dPR isolate showed reduced stability over the avian strains [73]. In vivo experiments were conducted with the 2UP_PA_eGFP_dPR H5N1 isolate exclusively. No signal came from live imaging, and only ex vivo lung images using IVIS showed a diffuse reporter signature, thus providing a model of avian influenza infection in mice [73].

To track vRNA movement in infected cells, a fluorescent tag can be fused to a viral protein involved in vRNA trafficking. Although some fluorescent IAV have been generated, most contain GFP fused with a viral protein not involved in vRNA transport or as a separate fluorescent polypeptide [71,89,90]. Lakdawala et al. overcame this limitation by fusing the entire GFP protein to the C-terminus of PA in the backbone of a WSN IAV (WSN-PA GFP) [87] (Figure 4D, Table 4). The segment constructed contained, after the GFP stop codon, a duplication of the PA 5′ packaging signals containing approximately 150 nt of the coding region, upstream of the 5′ NCR [87]. The authors developed two novel imaging tools: a system to visualize four different vRNA segments within an infected cell and a fluorescent influenza virus (WSN-PA GFP) to track vRNA dynamics in live cells during a productive infection [87]. Using the WSN-PA GFP virus and live cell fluorescence microscopy, the investigators were able to show PA-GFP foci fuse in the cytoplasm and remain in this state as they traveled to the plasma membrane [87]. Additionally, using fluorescent in situ hybridization probes for specific vRNAs, it was shown that PA-GFP fuses with PB2 and HA viral segments in the cytoplasm of infected cells [87]. Overall, the data suggested that vRNA segments are not exported as individual segments since the majority of foci at the external nuclear periphery contain more than one vRNA segment [87]. Moreover, many foci with fewer than 4 vRNA segments were observed in the cytoplasm, implying that all 8 vRNA segments are not exported from the nucleus together [87]. Therefore, the authors concluded that vRNA assembly includes the formation of flexible subcomplexes that export from the nucleus and then undergo further assembly *en route* to the plasma membrane via dynamic co-localization events [87].

### 2.4. Generation of Reporter-Expressing IAV Containing a Modified NA Segment

**Figure 5:**
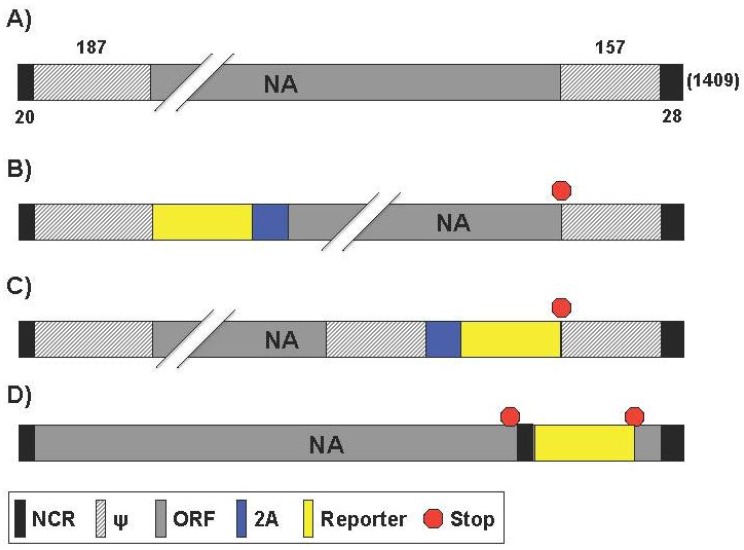
**NA reporter influenza A viruses:** Schematic representation of the NA segment from WT (**A**) and reporter (**B**–**D**) influenza A viruses as described in Figure 1. Nucleotide lengths for the NCR, ψ, and NA segment are indicated; (**B**,**C**) Bicistronic transcription of NA and reporter gene: Insertion of the 2A autocleavage site before (**B**) or after (**C**) the NA ORF separates the viral gene from the reporter gene. In (**C**), the packaging signals were duplicated before the 3′ NCR; (**D**) Dicistronic recombinant NA segment: The NA coding sequence is followed by a duplicated 3′ NCR, the reporter gene and the 5′ NCR.

Segment 6 from IAV (Figure 5A) encodes the NA protein that functions to promote viral release and is one of the major surface viral antigens [1]. Its principal biological role is the cleavage of the terminal sialic acid residues that are receptors for the HA glycoprotein [10,11]. The receptor-destroying activity in NA resides in the distal head domain that is linked to the viral membrane by an N-terminal hydrophobic transmembrane domain [91]. The ability to cleave sialic acid is also thought to help the virus penetrate mucus [92].

Feng et al. generated two eGFP-expressing PR8 viruses with the reporter linked to the NA segment [70] (Table 5). The NA vRNA packaging signals, including both the 3′ NCR (19 nt) and the adjacent 183 nt of the coding region; and the 5′ NCR (28 nt) and the adjacent 157 nt of the coding region, were maintained for the efficient packaging of the modified NA vRNA [70]. Each of the reporter PR8 viruses utilized the 2A autocleavage site to allow for collinear expression of both NA and eGFP [70]. In the “rPR8-eGFP+NA” virus, the eGFP gene precedes the 2A site, followed by NA (Figure 5B). In the “rPR8-NA+eGFP” the NA gene precedes eGFP and are separated by the 2A site (Figure 5C). What the authors found is that the order of viral gene and fluorescent reporter did have an effect on viral growth kinetics, plaque phenotype, NA activity, or eGFP localization. In embryonated eggs, MDCK, and human lung epithelial A549 cell lines, rPR8-NA-eGFP had a viral growth kinetic similar to WT virus, while rPR8-eGFP+NA replicated at lower levels (1 log or more) [70]. Likewise, and as expected, rPR8-NA+eGFP formed WT-like plaques that were large in comparison to rPR8-eGFP+NA [70]. Next, the amount of NA in rPR8-eGFP+NA and rPR8-NA+eGFP purified viruses were compared with those in WT virus. The rPR8-NA+eGFP virion possessed nearly as much NA activity (~90%) as the WT virus, while the rPR8-eGFP+NA virion possessed much less NA activity (~17%), which may be the result of eGFP interference and truncation of the N-terminal region of the NA protein [70]. In addition, NA vRNA packaging efficiency was tested. Compared to WT, nearly 80% of the NA+eGFP vRNA segments were packaged into the rPR8-NA+eGFP virions, while only 50% of eGFP+NA vRNA segments were packaged into the rPR8-eGFP+NA virions [70]. The localization of eGFP of the two recombinant viruses was also shown to be different. eGFP produced by rPR8-NA+eGFP was uniformly distributed throughout the infected cells, including the nucleus, while rPR8-eGFP + NA reporter was found only on the cell membrane [70]. The authors attribute the dissimilarities between viruses to the differences in the recombinant NA segments. rPR8-eGFP+NA has the reporter fused to the NA transmembrane anchoring region, thus localizing it to membrane. This could reduce the presence of NA on the membrane, limiting viral release. The authors suggest that this membrane bound eGFP would be useful for live infection monitoring in vivo but rPR8-eGFP+NA’s attenuated viral kinetics make rPR8-NA+eGFP the more logical choice [70]. However, replication-competent IAV harboring fluorescent reporters have been ineffective for such studies and only used ex vivo [38,39,41]. Bioluminescent harboring models have been far more effective for IAV in vivo research [39,46]. The authors also used the rPR8-NA+eGFP recombinant virus for the identification of neutralizing antibodies (NAbs) by fluorescence-activated cell sorting (FACS). FACS provides a rapid, highly accurate measurement of fluorescence measured by mean fluorescent intensity (MFI). Serial dilutions of mouse anti-PR8 serum were pre-incubated with rPR8-NA+eGFP and then the serum-fluorescent virus mixture was used to infect MDCK cells [70]. The authors showed incrementally increasing MFI, indicative of ineffective serum neutralization, in the lower concentration dilutions of serum, demonstrating the use of rPR8-NA+eGFP to evaluate the presence IAV NAbs.

Studying viral dissemination in vivo using replication-competent, reporter-expressing IAV remains a challenge because it requires that the reporter signal to be detected through tissue and skin. Pan et al. published the generation of replication-competent IAV harboring Gluc in the NA segment using PR8 as the backbone (IAV-Gluc) [72] (Table 5). The viral NA segment was similar to the described above for rPR8-NA + eGFP. IAV-Gluc replicated at lower levels in MDCK cells (2–3 log) and eggs (1–2 log) as compared with WT virus [72]. The authors were also able to visualize viral dissemination in live mice with a model virus that causes pathology. However, IAV-Gluc virus was attenuated in vivo; it took 1,000x more IAV-Gluc to achieve PR8 WT-like pathology as characterized by body weight change, percent survival, and lung histopathology [72]. Live-mouse imaging and antiviral therapeutic studies using IVIS was found to be optimal using a high dose (10^6^ PFU) for dissemination [72]. Importantly, Gluc signal was specific to the site of viral infection after the injection of the luciferase substrate. Finally, the authors tested whether IAV-Gluc can be used for evaluating antiviral therapeutics in vitro. As a proof-of-principle, they used an antiviral serum collected from convalescent mice previously infected with PR8. IAV-Gluc was pre-incubated with serial dilutions of the antiviral serum, the serum-virus mixture was used to infect MDCK cells in 96-well plates, and the bioluminescence intensity was determined at 24 h post-infection. Results showed that IAV-Gluc could be completely inhibited by the antiviral sera, demonstrating the potential of using IAV-Gluc for developing viral neutralization assays to evaluate antiviral drugs in vitro.

Vieira Machado et al. attempted the generation of a replication-competent IAV in the backbone of WSN harboring a dicistronic NA segment containing NA and foreign sequences with different sizes, either a foreign GFP (239 aa), chloramphenicol acetyl transferase (CAT; 220 aa) or a fragment of the *Mengovirus* VP0 capsid (101 aa) under the control of a duplicated 3′ promoter sequence (NA35-foreign gene) [93] (Table 5). To this end, the 3′ NCR and a multiple cloning site were inserted between the stop codon and the 5′ promoter sequence of the NA segment [93] (Figure 5D). Despite numerous attempts, a virus expressing GFP was not rescued. However, recombinant viruses expressing CAT or VP0 were successfully generated, suggesting that reporter genes other than GFP could be included and it can exist constraints on the size or the nature of the inserted foreign sequences. The authors demonstrated that the duplicated 3′ promoter was used to drive foreign gene expression [93]. Northern blot analysis for vRNA, cRNA and mRNA showed that two NA-derived RNA species were detected, corresponding with the full-length and a shorter subgenomic molecule comprising the reporter gene sequences flanked by 5′ and 3′ noncoding sequences [93]. Despite slightly reduced NA expression, the recombinant viruses replicated efficiently and proved to be stable upon serial passage in MDCK cells or in the pulmonary tissue of infected mice [93]. Later, Vieira Machado et al. generated a novel recombinant IAV (vNA38) harboring a dicistronic NA segment with an extended 5′ terminal sequence of 70 nts comprised of the last 42 nts of the NA ORF and the 5′ NCR [94] (Figure 5D, Table 5). vNA38 viruses, containing the same foreign genes as vNA35 viruses [93], replicated stably and more efficiently than vNA35 viruses with a dicistronic NA segment comprised of the native 5′ NCR only [94]. Moreover, whereas the NA35-GFP dicistronic vRNAs could not be rescued into infectious viruses, all three NA38-CAT, NA38-VP0 and NA38-GFP viruses were rescued [93]. In addition, vNA38 viruses expressed the foreign gene to higher levels than vNA35 viruses in cell culture and in the pulmonary tissue of infected mice [94]. The authors proposed this later recombinant IAV harboring the dicistronic NA segment for the development of live bivalent vaccines.

### 2.5. Generation of Reporter-Expressing IAV by Rearrangement of the PB1 and NS Viral Segment

**Figure 6:**
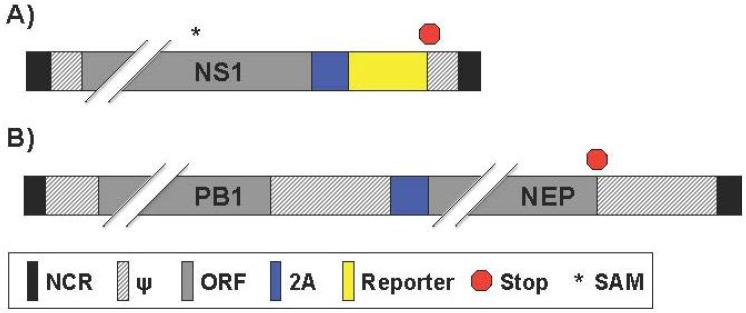
**Reporter influenza A viruses with genome rearrangement:** Schematic representation of mutant NS (**A**) and PB1 (**B**) viral segments as described in Figure 1. (**A**) Bicistronic transcription of NS1 and reporter gene: A splice acceptor mutation (SAM; *) inhibits alternative splicing. Reporter protein expression occurs after 2A cleavage; (**B**) Bicistronic transcription of PB1 and NEP: Expression of PB1 and NEP gene products occurs by insertion of the 2A autocleavage site sequence. PB1 3′ packaging signals were duplicated after the NEP ORF and before the 3′ NCR terminus.

Avian influenza virus subtypes H5N1 and H9N2 top the World Health Organization’s list for the greatest pandemic potential [95,96]. Inactivated H5N1 vaccines induce limited immune responses and, in the case of live-attenuated influenza vaccines (LAIV), there are safety concerns regarding the possibility of reassortment between the H5N1 viral segments and circulating IAV strains. To overcome these drawbacks, Pena et al. introduced a novel method of generating a bivalent vaccine against both influenza A/Guinea fowl/Hong Kong/WF10/1999 (H9N2) and influenza A/Vietnam/120320/04 (H5N1) using viral genome rearrangement [97] (Table 6). This was achieved by first removing NEP from the H9N2 NS viral segment. Then NEP was replaced with the H5 HA ORF separated by the FMDV 2A autocleavage site, allowing for collinear expression of both proteins (Figure 6A). The transgene was inserted by cloning it downstream of either a full-length or a truncated (expressing the first N-terminal 99 aa) *NS1* gene [97]. To prevent the normal splicing activity in the NS segment, the donor site and branch point within the full-length NS1 were mutated, and a stop codon was inserted early in the residual open reading frame of NEP. NEP was then fused to the H9N2 PB1 segment and separated by FMDV 2A (Figure 6B). In addition, the corresponding packaging signals previously determined for RNA segments 2 (PB1) and 8 (NS) were maintained at the 5′ end of each segment. As a proof of concept, the authors first used GFP or Gluc with the truncated NS1 ORF instead of the H5 HA viral protein and were able to rescue infectious, reporter expressing viruses (H9N2-GFP and -Gluc, respectively) [97]. These recombinant rearranged IAV reached titers on the order of six to seven log_10_ egg infectious dose (EID_50_)/mL, and transgene expression was maintained for up to ten passages [97]. A 10- to 100-fold reduction in virus titers of rearranged recombinant H9N2-GFP and H9N2-H5 HA (in the NS1-99aa backbone) was observed compared to parental recombinant virus (containing the same NS1 deletion) was observed [97]. In addition rearranged viruses were also attenuated in vivo. H9N2-GFP was not able to provide complete protection to mice after a single immunization. However, the H9N2-H5 HA virus provided complete protection against lethal challenge with influenza A/Vietnam/1203/2004 H5N1 in mice and ferrets, and also against a potentially pandemic H9:pH1N1 IAV reassortant virus [97,98]. Altogether, these studies, demonstrated that rearrangement of the IAV genome has great potential for the development of improved vaccines against multiple IAV, as well as other pathogens, and for the expression of reporter genes [97].

The genome rearrangement strategy was also extended to influenza A/California/04/2009 (pH1N1 or Ca04) by Sutton et al. where Gluc or GFP were expressed downstream of NS1 ORF (designated GlucCa04 and GFPCa04, respectively) [99] (Table 6). The researchers also rescued an amantadine-resistant GlucCa04 virus for anti-viral drug screening purposes and denoted (Res/GlucCa04) [99]. GlucCa04 and Res/GlucCa04 grew to significantly reduced titer levels compared to the recombinant Ca04 and Res/Ca04 WT counterparts in MDCK cells over multicycle growth [99]. Both the GlucCa04 and Res/GlucCa04 grew to similar titers, although the sensitive virus appears to grow to slightly higher titers than the resistant virus [99]. The genetic stability of three separate replicates of GlucCa04 was evaluated by serial passaging five times in MDCK cells. Of the three replicates, two had a ten-fold decrease in Gluc expression while one had a ten-fold increase in reporter expression after passaging, although the cause for these differences was not further explored. As a proof-of-concept, Sutton et al. used these viruses for an in vitro anti-viral screening microneutralization assay. Amantadine treatment significantly decreased Gluc expression of GlucCa04 compared to Res/GlucCa04, with half maximal inhibitory concentration (IC_50_) results comparable to previously published literature. Microneutralization assay outcomes also showed similar results between pH1N1 WT and GlucCa04 [99]. Finally, the authors showed that GlucCa04 could be used as an in vivo screening tool for compounds with antiviral activity [99]. When taken all together, GlucCa04 provides another luciferase-based tool to use as a screening technique to identify novel antiviral drugs, shortening the time required for virus detection for in vitro and in vivo studies.

### 2.6. Generation of Reporter-Expressing, Replicating-Competent IAV Containing a Recombinant NS Segment Harboring the Foreign Gene

**Figure 7:**
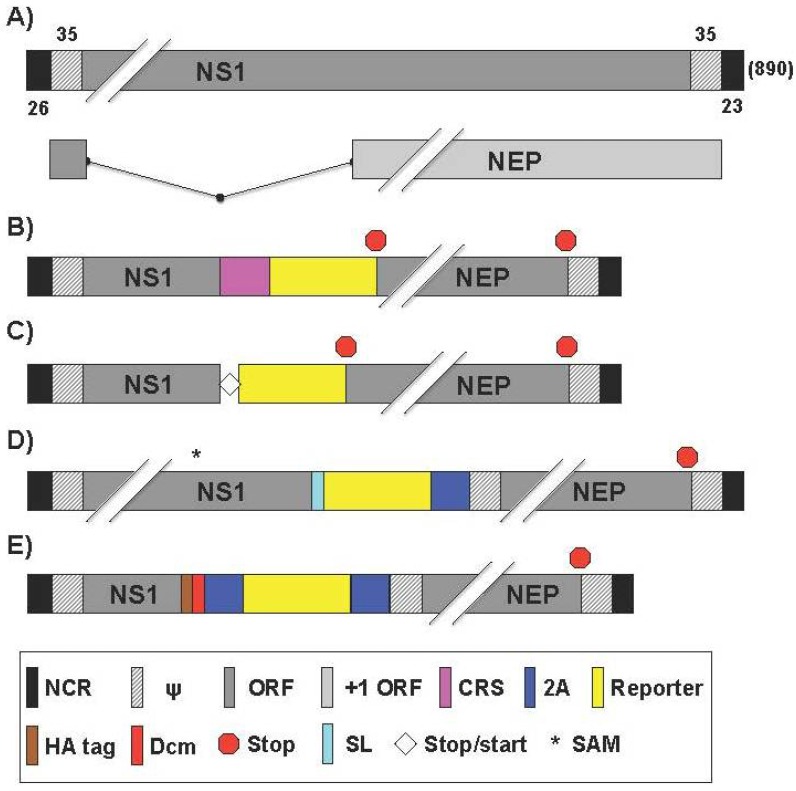
**NS reporter influenza A viruses:** Schematic representation of the NS segment from WT (**A**) and reporter (**B**–**E**) influenza A viruses as described in Figure 1. Influenza A WT NS gene segment encodes for NS1 and NEP via alternative splicing. Nucleotide lengths for the NCR, ψ, and NS segment are indicated; (**B**) Multicistronic transcription using a caspase recognition site: Multicistronic NS reporter influenza A viruses were generated by insertion of a caspase recognition site (CRS) after the NS1 ORF; (**C**) Multicistronic transcription using stop-start sequence: Multicistronic NS reporter influenza A viruses were generated by insertion of a stop/start transcription site after the NS1 ORF for independent translation of NS1, reporter gene, and NEP; (**D**) NS1 fusion protein: Reporter genes were fused to native NS1 ORF with a short linker (SL). A splice acceptor mutation (SAM; *) inhibits NEP alternative splicing. NEP expression occurs after 2A cleavage. The 5′ ψ are duplicated and contain NEP N-terminal amino acid codons; (**E**) Tricistronic transcription of the NS segment: Reporter gene and NEP expression occurs after two 2A cleavage sites. The 5′ ψ are duplicated and contain NEP N-terminal amino acid codons. An HA tag and a heterologous dimerization domain (Dcm) were added after the NS1 ORF.

Research using a replication-competent IAV containing a modified NS segment (Figure 7A) started as early as 2004. Kittel et al. published a study where GFP was fused to a truncated (only expressing the first 125 aa) NS1 protein separated by a peptide sequence that contained a caspase recognition site (CRS) [90] (Figure 7B, Table 7). The truncated NS1 maintained the ability to antagonize type I interferon (IFN) and allowed for the insertion of a foreign gene, like GFP. The GFP-harboring IAV (NS1-GFP) was able to replicate in protein kinase R (PKR) knockout mice and reached similar viral lung titers with or without the GFP gene (5 × 10^4^ PFU/g), but were attenuated in WT mice [90]. In vitro, NS1-GFP was capable of replicating to WT-like titers in IFN-deficient Vero cells [90]. In contrast to Vero, virus passaged in IFN-competent MDCK cells resulted in the selection of NS1-GFP deletion mutants [90]. Sequence data of these mutants confirmed that loss of GFP expression correlated with partial deletion of the GFP ORF. The deletions were very heterogeneous, ranging from several nucleotides up to the removal of approximately 80% of the C terminal end of the GFP protein. Interestingly, the deletion mutants that outcompeted NS1-GFP only contained GFP deficiencies and not NS1 mutations. While certainly not the most practical replication-competent, fluorescent-expressing IAV, Kittel et al. provided an early, efficacious genetic strategy for inserting foreign genes into a viral genome that yielded viable progeny [90].

Kittel et al. also incorporated genetic characteristics of influenza B virus (IBV), which uses a unique strategy to encode the matrix 2 (BM2) protein [107]. The initiation codon of the BM2 overlaps with the termination codon of the upstream gene for the M1 protein, forming a stop-start pentanucleotide (UAAUG) [107]. Utilizing the IBV strategy, Kittel et al. developed a bicistronic recombinant IAV in which the stop codon of the stop-start cassette terminates the translation of NS1 after 125 aa and the start codon reinitiates the translation of GFP (A/PR8/NS1-GFPStSt) (Figure 7C, Table 7) [100]. Although the expression level of GFP was significantly lower than that obtained previously with the NS1-GFP [90], the bicistronic IAV appeared to be replication competent in mice and showed higher genetic stability. In fact, all viral isolates derived from infected mouse lungs were still capable of expressing GFP in infected cells [100]. Utilizing this bicistronic virus, authors also expressed interleukin-2 (IL-2) instead of GFP [100]. Although the IL-2-expressing IAV showed high titers in mouse lungs, it did not display any mortality rate in infected animals. In addition, the IL-2-expressing virus showed an enhanced CD8+ response to viral antigens in mice after a single intranasal immunization. These results suggested that influenza viruses could be engineered for the expression of biologically active molecules such as cytokines for immune modulation purposes [100].

Manicassamy et al. described a replication-competent IAV that contained GFP fused to NS1 protein (NS1-GFP) of PR8 [71]. It had been previously shown that functionally active IAV NS1 and NEP could be expressed as a single polyprotein with a FMDV 2A autoproteolytic cleavage site [108]. Manicassamy et al. modified the NS segment to express NS1-GFP and NEP as a single polyprotein with a porcine teschovirus-1 (PTV-1) 2A autoproteolytic cleavage site between them, allowing NEP to be released from the upstream NS1-GFP protein during translation (Figure 7D, Table 7) [71]. In order to avoid the splicing of NS mRNA, two silent mutations in the splice acceptor site were introduced [71]. NS1-GFP replicated like WT in a single-cycle replication assay in MDCK cells but showed 100x lower replication levels in a multicycle assay [71]. A well-characterized function of IAV NS1 protein is to counteract the host type I IFN response [20]. It was shown that NS1-GFP was also able to suppress IFN activity. In vivo, NS1-GFP was 100x attenuated when evaluated by MLD_50_ and body weight loss compared to WT virus [71]. Using the NS1-GFP virus, the authors studied viral dissemination in lungs. Not only was GFP observable in the lungs of infected mice ex vivo using IVIS for whole-organ imaging, but could also be observed using flow cytometry to analyze the infection progression in antigen presenting cells [71]. Imaging of murine lungs showed that infection starts in the respiratory tract in areas close to large conducting airways and later spreads to deeper sections of the lungs [71]. The authors found that using a 10^6^ PFU intranasal inoculation, 10% of dendritic cells (DCs) were observed to express GFP and 2%–3% of macrophages and neutrophils were also GFP+ at 48 h post-infection [71]. At 96 h, the percent of GFP-expressing DCs declined but percentages of fluorescing macrophages and neutrophils increased [71]. Dosing animals with oseltamivir limited GFP expression in all antigen-presenting cells while amantadine was only effective in specific cell types [71]. The authors do however acknowledge that antigen presenting cells could be GFP+ due to phagocytizing infected cells. In fact, Helft et al. used the NS1-GFP IAV to establish the kinetics of infection and transport to the draining lymph nodes [109] (Table 7). The authors provided evidence that lung DCs that transport viral antigens to the draining lymph nodes are protected from influenza virus infection in vivo and that induction of viral-specific CD8+ T cell immunity is mainly dependent on cross-presentation of virally infected cells by lung migratory non-infected CD103^+^ DCs [109]. They also reported that lung migratory CD103+ DCs express a natural anti-viral state that is further strengthened by type I IFN released during the first few hours following influenza virus infection.

This NS1-GFP has been also used in other studies. For instance, Hufford and Richardson et al. utilized NS1-GFP to demonstrate that lung-resident neutrophils are infected by IAV [110] (Table 7). The authors suggest that they can act not only to stimulate the innate immune response, but to also activate CD8+ T lymphocytes to begin viral clearance in the lung. While the innate immune system is critical for antiviral clearance, its signaling can promote IAV replication. A study by Pang et al. has shown that innate immune activation triggered by toll-like receptor 7 (TLR7) and retinoic acid inducible gene-1 (RIG-I) is required for efficient IAV replication in the reparatory tract [102] (Table 7). NS1-GFP was utilized here by infecting bone marrow-derived DCs (BMDCs) that are deficient in genes required for TLR7 and RIG-I signalling. Consistent with previous studies [111], WT BMDCs were less susceptible to infection than TLR7 and RIG-I deficient cells as determined by GFP fluorescence. Comparison of IAV-infected cells showed that inflammatory mediators elicited by TLR7 and RIG-I signaling recruit viral target cells to the airway, thereby increasing viral load within the respiratory tract. The authors suggested that IAV uses physiological levels of inflammatory responses to its replicative advantage, highlighting the complex interplay between viruses and the host innate-immune responses [102].

A study by Resa-Infante et al. showed that without importin-α7, NS1-GFP is unable to efficiently replicate in the alveolar epithelium [103] (Table 7). Direct observation of IAV infection provides a means to evaluate antiviral therapies. In a study by Kim et al., NS1-GFP infected cells were treated with an NA inhibitor (oseltamivir) and various natural compounds [112] (Table 7). Oseltamivir was also used in vivo by treating infected mice [112]. In both cases, reduced GFP expression was indicative of limited or neutralized infection.

Mice are commonly used to model IAV infection in vivo, but Gabor et al. have reported IAV infection in zebrafish as a new and inexpensive model to reproduce viral infection [101] (Table 7). Using IAV PR8 (H1N1), X31 (H3N2) and NS1-GFP, they showed that zebrafish can support infection and mount an immune response [101]. Interestingly, infected (GFP+) cells were localized within the cardiovascular system and the swim bladder [101]. The authors point out that the swim bladder can be likened to IAV infection in human lung endothelial tissue, further supporting the relevance of the model.

Due to technical limitations, it has been historically difficult to monitor the process of IAV infection. Modern fluorescent microscopy has given researchers the ability to use video to capture infection of fluorescent-expressing, replication-competent IAV. Using NS1-GFP, Roberts et al. demonstrated that IAV-infected cells can infect neighbouring cells by passing the virus through actin protrusions [104] (Table 7). Previous observations using NA-deficient viruses [36], and treating infected cells with NA inhibitors by Roberts et al. [104], showed that microplaques of IAV appear in cell culture [104]. However, the addition of actin inhibitors or microtubule stabilizers to oseltamivir treatment prohibited microplaque formation [104]. Live video microscopy shows compelling evidence for cell-to-cell IAV infection, NS1-GFP-infected cells show GFP movement from one cell to another through an intercellular connection [104]. This, coupled with immunofluorescence data showing actin filaments containing vRNPs connecting infected to uninfected cells, led to the hypothesis that cell-free virion infection is not the only method of IAV dissemination [104].

Using a similar NS1 construct to NS1-GFP, Eckert et al. generated IAV encoding maxGFP, turboRFP, or Gluc that replicated comparably to WT PR8 [48] (Table 7). The researchers moved forward only with NS1-Gluc because it showed the greatest reporter stability of the three viruses, while NS1-maxGFP and -turboRFP dropped below 5% fluorescent positive cells on the third passage in tissue culture [48]. Since Gluc is secreted, it is well suited as a reporter for high throughput antiviral compound screening. After establishing a correlation between the viral titer and luciferase activity, zanamivir (an IAV NA inhibitor) was used to benchmark NS1-Gluc in infected human epithelial colorectal adenocarcinoma CaCo-2 cells [48]. Numerous antiviral factors are expressed upon type I IFN induction. Members of the IFN-inducible transmembrane (IFITM) protein family, specifically IFITM1, 2 and 3, have been identified as inhibitors of IAV [113]. The authors showed that NS1-Gluc virus can be used to investigate cellular proteins that exhibit inhibitory functions against IAV infection [48]. Remarkably, IFITM2 and 3 proteins, when transduced into A549, MDCK, and 293T cells, inhibit the proliferation of IAV [48]. In contrast, the inhibitory effects observed upon expression of IFITM1 were minor (A549 and human embryonic kidney 293T cells) or absent (MDCK cells), again in agreement with published data [113,114]. Therefore, the data indicated that NS1-Gluc virus could be used to evaluate the antiviral activity of host cell proteins [48]. In fact, previous studies have suggested that the IFITM proteins act by increasing endosomal cholesterol [115]. The authors found that U18666A, a compound that increases endosomal cholesterol [116], displayed a dose dependent inhibitory effect against IAV infection in vitro [48].

Likewise, Nogales et al. used a similar approach to NS1-GFP to generate an IAV expressing the monomeric (m)Cherry fluorescent protein in the backbone of PR8 or pH1N1 (PR8- and pH1N1-mCherry, respectively) [41] (Table 7). PR8-mCherry replicated at lower levels than WT in MDCK cells [41]. However, the PR8-mCherry virus was able to abrogate type I IFN induction similarly to PR8 WT [41]. Importantly, both mCherry-expressing viruses were inhibited with antivirals or type-specific NAbs to levels comparable to WT viruses, representing an excellent option for the rapid identification of antivirals or NAbs using HTS methodologies [41]. The authors also presented the potential use for PR8-mCherry for in vivo studies [41]. Even though PR8-mCherry was attenuated in mice, inoculation with 10^4^ PFU resulted in infection-specific fluorescence, and replication could be directly visualized and quantified from whole excised lung using IVIS [41]. These results offer a promising option to directly study the biology of IAV and to evaluate experimental outcomes from treating IAV infections in vitro and ex vivo [41].

The reporter-expressing IAV discussed thus far all encode static reporters, which are not optimal for determining the origin or chronology of infection. A dynamic fluorescent protein Timer was engineered that changes its emission spectra from green to red over time and could allow for tracking IAV in more detail [117]. Timer is derived from the red fluorescent protein of *Discosoma* (DsRed) and contains two point mutations that confer a strong quantum yield and the spectral shift phenotype [117]. Breen et al. described the generation of replication-competent viruses expressing Timer fused to the viral protein NS1 in the backbone of pH1N1 (IAV-Timer) and influenza B/Brisbane/60/2008 (IBV-Timer) viruses [68] (Table 7). The recombinant IAV-Timer, in vitro, showed similar growth kinetics compared to the WT virus [68]. Using multiple approaches, including fluorescent microscopy and plaque assays, the authors were able to differentiate primary from secondarily infected cells [68]. Timer expression and spectral shift were quantified in infected cells using a fluorescence plate reader and flow cytometry. Importantly, IAV-Timer was useful to evaluate the dynamics of viral infections in mouse lungs using IVIS [68]. These studies constitute proof-of-principle of the usefulness for recombinant IAV expressing the dynamic Timer protein to study viral infection dynamics both, in vitro and in vivo [68].

A drawback of many fluorescent or luminescent IAV is that they are commonly attenuated in vivo [38,39,41]. Fukuyama et al. generated a series of Color-flu viruses in the backbone of PR8, expressing fluorescent proteins of different colors: Venus, eCFP, mCherry and eGFP [38] (Table 7). Fukuyama et al. then mouse-adapted (MA) each of the PR8 reporter-expressing IAV by serial passaging in mice [38]. The MA virus variants (MA-PR8-eGFP, -eCFP, -Venus, and -mCherry) showed higher pathogenicity compare to parental viruses, although they were still less pathogenic than WT PR8 as determined by MLD_50_ [38]. The ability to use the four unique viruses for multiplex was tested in infected mice. Each reporter could be observed in clusters of whole-lung explants using stereomicroscopy. Additionally, co-infection could be observed in lung tissue slices from viruses containing two or more fluorescent reporters [38]. This suggests that Color-flu can be used to study reassortment, which is implicated in the generation of pandemic strains of IAV along with the generation of human adapted strains [22,24,118,119]. The researchers also applied MA-PR8-Venus to analyze the macrophage response to IAV infection [38]. Macrophages were observed to infiltrate the bronchial epithelium around MA-PR8-Venus positive cells. Transcriptome analysis of infected (Venus-positive) macrophages showed elevated message levels of genes involved in innate immune response [38]. In addition, a HPAI virus was engineered to contain Venus (MA-HPAI-Venus). Mice infected with MA-HPAI-Venus resulted in a greater Venus-positive bronchial epithelium than MA-PR8-Venus as observed by two-photon microscopy [38]. There were also increased numbers of Venus-positive macrophages in MA-HPAI-Venus infections, supporting findings that H5N1 HPAI viruses induce more severe inflammatory responses in the lung of infected mice than PR8 [38]. Therefore, these studies demonstrated the utility of Color-flu for comparative studies of IAV pathogenesis [38].

Most of the recombinant IAV discussed use a single 2A autocleavage site to collinearly express an NS1-reporter fusion protein and NEP separately, without the need for alternate splicing [38,39,41,48,52,66,68,69,70,71,72]. Recently, De Baets et al. have reported the generation of an IAV expressing GFP from a tri-cistronic NS segment in the backbone of PR8 (Figure 7E, Table 7) [105]. To reduce the size of this engineered gene segment, they used a truncated NS1 protein of 73 aa combined with a heterologous dimerization domain of the *Drosophila melanogaster* nonclaret disjunctional (Ncd) protein [120], to increase protein stability. GFP and NEP sequences were in frame after the truncated NS1, and were each separated by 2A self-processing sites (from FMDV and PTV-1, respectively) [105]. An HA-tag was also fused to NS1 to facilitate protein detection. The resulting PR8-NS1(1–73)GFP virus replicated as efficiently as PR8 WT in vitro and retained reporter expression in 100% of plaques from 5 passages in MDCK-V cells, which have their IFN response blocked by stable expression of parainfluenza virus type 5 V protein [105]. However, when passaged in parental MDCK cells, only 23% of plaques were GFP-positive after five passages [105]. The recombinant virus was slightly attenuated in vivo but maintained 96.4% GFP-positive plaques when recovered five days post-infection from mouse lungs [105]. The cellular tropism of PR8-NS1(1–73)GFP was also evaluated during treatment with either oseltamivir or a MAbs targeted against the IAV matrix protein 2 ectodomain (M2e) [105]. The latter treatment is of interest based on data that show efficacy of M2e IAV vaccines [121]. Both treatments protected mice from weight loss post PR8-NS1(1–73)GFP infection [105]. Finally, the authors demonstrated the usefulness of this virus to study the viral cell tropism ex vivo. The prophylactic treatment of mice with anti-M2e MAb or oseltamivir resulted in a decrease in the percentage of GFP-expressing cells [105]. Future studies must address if resistance to this MAb through antigenic mutation of the M2 protein could nullify this treatment.

Reuther et al. also have reported the generation of IAVs expressing GFP from a tri-cistronic NS segment in the backbone of A/SC35M (H7N7) (Figure 7E, Table 7) [106]. The resulting viruses encoding luciferases or fluorescent proteins maintained high genetic stability in vitro up to 4 rounds of passaging in human cells, and were characterized in vivo. Therefore, the recombinant viruses generated could be readily employed for antiviral compound screenings in addition to visualization of infected cells or cells that survived acute infection.

## 3. Conclusions and Future Directions

The purpose of this review is to discuss the biology and applications of replication-competent IAV expressing the most commonly used fluorescent or luciferase reporter genes. Plasmid-based reverse genetics techniques allow for the simultaneous expression of the IAV RNA-dependent RNA polymerase (RdRp) and negative-stranded genome viral segments in transiently transfected mammalian cells, which together generate de novo, or rescue, recombinant IAV [122,123]. Moreover, these techniques allowed the use of recombinant DNA technology to modify the genome of IAV and to engineer viruses expressing foreign genes. Recombinant reporter-expressing, replicating-competent IAV are applicable to translational research and have been demonstrated in screening platforms to identify specific or broadly reactive NAbs, antiviral compounds, or host proteins involved in viral replication. In fact, the generation of recombinant, reporter-expressing IAV lead the design of cell-based assays that capture all stages of the virus life-cycle. With these replicating-competent reporter viruses, there is greater flexibility in the choice of cells to perform these assays, providing increased potential for identifying inhibitors of both viral and cellular functions that are critical for optimal virus replication. Moreover, these fluorescent- or luciferase-expressing IAV have been shown to be a valuable asset for in vivo studies when used in conjunction with new imaging technologies. The combination of fluorescent or luminescent genes plus the generation of reporter IAV using different backbones, including HPAI or pandemic strains, have increased the spectrum of tools that can be used to facilitate the study of these IAV. Other future areas of replication-competent IAV application include, but are not limited to, virus tropism and the analysis of viral reassortments during replication or vaccine development. In conclusion, there are potential advantages and disadvantages associated with replication-competent IAV; however, the number of applications as well as the abundant economic, quantitative, and biological advantages of these IAV, highlight their promising applications in basic and translational influenza research in the imminent future.

## Figures and Tables

**Table 1:** Fluorescence versus bioluminescence, features and applications.

Properties	Fluorescence	Bioluminescence
Enzymatic amplification of signal	NO	YES
Substrate required for assay	NO	YES
High Reproducibility	YES	YES
FACS-compatible	YES	NO
In vitro applications	YES	YES
Ex vivo applications	YES	YES
In vivo applications	NO	YES
Flexible readout	YES	NO
Analysis of individual cells	YES	NO
HTS	YES	YES
Analysis of intermolecular interactions	YES	NO
Detection	Fluorescence	Luminescence

FACS: fluorescence-activated cell sorting; HTS: High-Throughput Screening.

**Table 2:** Influenza A viruses with reporter genes in the PB2 viral segment.

Gene	Virus Backbone ^(1)^	Transgene ^(2)^	Insertion Mechanism ^(3)^	Application	Ref.
*PB2*	WSN	Split GFP	Fusion	Virus Biology	[58,61]
*PB2*	PR8, WSN	Gluc	2A site	Neutralizing antibodies Antivirals	[66,67]

^(1)^ WSN: A/WSN/1933 (H1N1); PR8: A/Puerto Rico/8/1934 (H1N1); ^(2)^ GFP: Green fluorescent protein; Gluc: Gaussia luciferase; ^(3)^ Fusion: The C-terminal region of the reporter was fused to PB2; 2A site: The reporter gene was separated from the viral ORF via a 2A peptide sequence.

**Table 3:** Recombinant influenza A viruses expressing reporter gene in the viral PB1 segment.

Gene	Virus Backbone ^(1)^	Transgene ^(2)^	Insertion Mechanism ^(3)^	Application	Ref.
*PB2* *PB1* *PA*	WSN	Split Gluc	Fusion	Virus-host interaction	[80]

^(1)^ WSN: A/WSN/1933 (H1N1); ^(2)^ Gluc: Gaussia luciferase; ^(3)^ Fusion: A fragment of a split Gluc (Gluc1 or Gluc2) was fused to the C-terminus of PB1, PB2, or PA. To reconstitute the Gluc activity, both fragments needs to be in the same cell.

**Table 4:** Reporter-expressing recombinant influenza viruses in the viral PA.

Gene	Virus Backbone ^(1)^	Transgene ^(2)^	Insertion Mechanism ^(3)^	Application	Ref.
*PA*	WSN	Nluc	2A site	Virus biology and transmission	[50,52]
*PA*	pH1N1	Nluc	2A site	Virus biology and transmission	[69]
*PA*	PR8, Neth602, Ind5, Anh1	eGFP, fRFP, iRFP, Gluc, FFluc	2A site	Virus biology	[73]
*PA*	WSN	GFP	Fusion	Virus biology	[87]

^(1)^ WSN: A/WSN/1933 (H1N1); pH1N1: A/California/04/2009 (pH1N1); PR8: A/Puerto Rico/8/1934 (H1N1); Neth602: A/Netherlands/602/2009 (H1N1); Ind5: A/Indonesia/5/2005 (H5N1); Anh1: A/Anhui/1/2013 (H7N9); ^(2)^ Nluc: Nanoluciferase; eGFP: Enhanced GFP; Gluc: Gaussia luciferase; fRFP: far-red fluorescent protein; iRFP: near-infrared fluorescent protein; FFluc: Firefly luciferase; GFP: Green fluorescent protein; ^(3)^ 2A site: The reporter gene was separated from the viral ORF via a 2A peptide sequence; Fusion: Fusing the entire GFP protein to the C-terminus of PA.

**Table 5:** NA recombinant reporter-expressing influenza A viruses.

Gene	Virus Backbone ^(1)^	Transgene ^(2)^	Insertion Mechanism ^(3)^	Application	Ref.
*NA*	PR8	eGFP	2A site	Virus biology	[70]
*NA*	PR8	Gluc	2A site	Virus biology	[72]
*NA*	WSN	GFP	Viral promoter	Virus biology	[93,94]

^(1)^ PR8: A/Puerto Rico/8/1934 (H1N1); WSN: A/WSN/1933 (H1N1); ^(2)^ eGFP: Enhanced GFP; Gluc: Gaussia luciferase; GFP: Green fluorescent protein; ^(3)^ 2A site: The reporter gene was separated from the viral ORF via a 2A peptide sequence; Viral promoter: The reporter was introduced under the control of a duplicated 3′ NCR (viral promoter).

**Table 6:** Reporter-expressing recombinant influenza A viruses with a rearranged genome.

Gene	Virus Backbone ^(1)^	Transgene ^(2)^	Insertion Mechanism ^(3)^	Application	Ref.
NS	HK99 VN1203	GFP, Gluc	Genome rearrangement	Vaccine	[97]
NS	pH1N1	GFP, Gluc	Genome rearrangement	Antivirals	[99]

^(1)^ HK99: A/Guinea Fowl/Hong Kong/WF10/1999 (H9N2); VN1203: A/Vietnam/1203/2004 (H5N1); pH1N1: A/California/04/2009 (H1N1); ^(2)^ GFP: Green fluorescent protein; Gluc: Gaussia luciferase; ^(3)^ Genome rearrangement: The NEP was removed from the NS viral segment, and replaced with the foreign gene separated by a 2A autocleavage site. Then, the NEP was fused to the PB1 segment also separated by a 2A site.

**Table 7:** Recombinant influenza A viruses expressing reporter genes from the NS viral segment.

Gene	Virus Backbone ^(1)^	Transgene ^(2)^	Insertion Mechanism ^(3)^	Application	Ref.
*NS*	PR8	GFP	Caspase recognition site	Virus biology	[90]
*NS*	PR8	GFP	Stop/start	Vaccine	[100]
*NS*	PR8	maxGFP	2A site	Virus pathogenesis	[71,101,102,103,104]
*NS*	PR8	maxGFP, turboRFP, Gluc	2A site	Antiviral and virus-host interaction	[48]
*NS*	PR8 pH1N1	mCherry	2A site	Antivirals, neutralizing antibodies, virus pathogenesis	[41]
*NS*	pH1N1	Timer	2A site	Virus propagation	[68]
*NS*	PR8 VN1203	Venus, eGFP, eCFP, mCherry	2A site	Virus-host interaction and virus pathogenesis	[38]
*NS*	PR8 WSN	GFP	2× 2A site	Virus pathogenesis	[105,106]

^(1)^ PR8: A/Puerto Rico/8/1934 (H1N1); pH1N1: A/California/04/2009 (H1N1); VN1203: A/Vietnam/1203/2004 (H5N1); ^(2)^ GFP: Green fluorescent protein; maxGFP: advanced version of eGFP; Gluc: Gaussia luciferase; mCherry: monomeric Cherry fluorescent protein; Timer: modified Discosoma red fluorescent protein; Venus: advanced version of yellow fluorescent protein; eGFP: Enhanced GFP; eCFP: Enhanced cyan fluorescent protein; ^(3)^ Caspase recognition site: The reporter gene was fused to NS1 protein separated by a peptide sequence containing a caspase recognition site; Stop/Start: The stop-start pentanucleotide (UAAUG) from BM2 of influenza B virus was inserted between NS1 and the reporter gene; 2A site: The reporter gene was separated from the viral ORF via a 2A peptide sequence.

## Abbreviations

WSN	A/WSN/1933 H1N1
PR8	A/Puerto Rico/8/1934
pH1N1	A/California/04/2009 H1N1
Neth602	A/Netherlands/602/2009 H1N1
Ind5	A/Indonesia/5/2005 H5N1
Anh1	A/Anhui/1/2013 H7N9
HK99	A/Guinea Fowl/Hong Kong/WF10/1999 H9N2
VN1203	A/Vietnam/1203/2004 H5N1
maxGFP	advanced version of eGFP
Venus	advanced version of yellow fluorescent protein
aa	amino acid
2A	autocleavage 2A site
BMDCs	bone marrow-derived DCs
CRS	caspase recognition site
CAT	chloramphenicol acetyl transferase
DCs	dendritic cells
dPR	duplicated packaging region
EM	electron microscopy
ER	endoplasmic reticulum
eCFP	enhanced cyan fluorescent protein
eGFP	enhanced green fluorescent protein
fRFP	far-red fluorescent protein
FFluc	firefly luciferase
FACS	fluorescence-activated cell sorting
FMDV	foot-and-mouth disease virus
Gluc	Gaussia luciferase
GFP	green fluorescent protein
HA	hemagglutinin
HTS	high-throughput screening
IAV	influenza A virus
BM2	influenza B virus M2 protein
IL-2	interleukin-2
IVIS	in vivo imaging system
MDKC	Madin-Darby canine kidney epithelial cells
M1	matrix 1 protein
M2	matrix 2 protein
M2e	matrix 2 protein ectodomain
MFI	mean fluorescent intensity
Timer	modified DsRed fluorescent protein Timer
MAbs	monoclonal antibodies
mCherry	monomeric cherry fluorescent protein
MLD_50_	mouse lethal dose-50
NLuc	NanoLuc
iRFP	near-infrared fluorescent protein
NA	neuraminidase
NAb	neutralizing antibody
Ncd protein	nonclaret disjunctional protein
NCRs	non-coding regions
NS1	non-structural protein 1
NEP	nuclear export protein
NP	nucleoprotein
nt	nucleotide
ORF	open reading frame
ψ	packaging signals
PFU	plaque forming units
PTV-1	polymerase acidic (PA) and basic (PB1 and PB2) subunits, porcine teschovirus-1
PET/CT	positron emission tomography-computed tomography
PKR	protein kinase R
DsRed	red fluorescent protein of Discosoma
RIG-I	retinoic acid inducible gene-1
RdRp	RNA-dependent RNA polymerase
SL	short linker
SAM	splice acceptor mutation
TCID_50_	tissue culture infectious dose 50
TLR7	toll-like receptor 7
IFN	type I interferon
IFITM proteins	type I IFN-induced transmembrane proteins
UTR	untranslated region
vRNA	viral RNA
vRNP	viral ribonucleoprotein
WT	wild-type
